# The structural basis for Z α_1_-antitrypsin polymerization in the liver

**DOI:** 10.1126/sciadv.abc1370

**Published:** 2020-10-21

**Authors:** Sarah V. Faull, Emma L. K. Elliston, Bibek Gooptu, Alistair M. Jagger, Ibrahim Aldobiyan, Adam Redzej, Magd Badaoui, Nina Heyer-Chauhan, S. Tamir Rashid, Gary M. Reynolds, David H. Adams, Elena Miranda, Elena V. Orlova, James A. Irving, David A. Lomas

**Affiliations:** 1 UCL Respiratory, University College London, 5 University Street, London WC1E 6JF, UK.; 2 Institute of Structural and Molecular Biology, University College London, Gower Street, London WC1E 6BN, UK.; 3 Leicester Institute of Structural and Chemical Biology, University of Leicester, Henry Wellcome Building, Lancaster Road, Leicester LE1 7HB, UK.; 4 National Institute for Health Research (NIHR) Leicester BRC-Respiratory, Leicester, UK.; 5 Institute of Structural and Molecular Biology, Birkbeck College, Malet Street, University of London, London WC1E 7HX, UK.; 6 Centre for Stem Cells and Regenerative Medicine and Institute for Liver Studies, King’s College London, London WC2R 2LS, UK.; 7 Centre for Liver Research and NIHR Birmingham Liver Biomedical Research Unit, Institute of Immunology and Immunotherapy, University of Birmingham, Birmingham, UK.; 8 Department of Biology and Biotechnologies “Charles Darwin” and Pasteur Institute—Cenci Bolognetti Foundation, Sapienza University of Rome, Rome, Italy.

## Abstract

The serpinopathies are among a diverse set of conformational diseases that involve the aberrant self-association of proteins into ordered aggregates. α_1_-Antitrypsin deficiency is the archetypal serpinopathy and results from the formation and deposition of mutant forms of α_1_-antitrypsin as “polymer” chains in liver tissue. No detailed structural analysis has been performed of this material. Moreover, there is little information on the relevance of well-studied artificially induced polymers to these disease-associated molecules. We have isolated polymers from the liver tissue of Z α_1_-antitrypsin homozygotes (E342K) who have undergone transplantation, labeled them using a Fab fragment, and performed single-particle analysis of negative-stain electron micrographs. The data show structural equivalence between heat-induced and ex vivo polymers and that the intersubunit linkage is best explained by a carboxyl-terminal domain swap between molecules of α_1_-antitrypsin.

## Teaser

α_1_-Antitrypsin polymers from human tissue have a structure most consistent with an intermolecular C-terminal domain swap.

## INTRODUCTION

The misfolding of proteins and their spontaneous ordered aggregation underlie the pathology of Alzheimer’s, Huntington’s, and Parkinson’s diseases; amyloidoses; and serpinopathies—the latter involving self-association of mutant members of the serine protease inhibitor (serpin) superfamily. α_1_-Antitrypsin is a 52-kDa serpin expressed and secreted predominantly by hepatocytes and is the most abundant circulating protease inhibitor. The primary physiological role of α_1_-antitrypsin is the inhibition of neutrophil elastase, a protease whose production is increased during the acute phase inflammatory response (fig. S1, A and B). However, genetic variants such as the severe Z (E342K) allele of α_1_-antitrypsin promote proteasomal degradation and the formation of ordered linear polymers (*1*, *2*). Despite the pronounced retention in the endoplasmic reticulum (ER), α_1_-antitrypsin polymers do not typically initiate the unfolded protein response. Instead, these ordered aggregates can be sequestered into ER-derived inclusion bodies that are associated with liver disease. The lack of circulating α_1_-antitrypsin results in dysregulation of neutrophil elastase and hence tissue destruction and emphysema (*2*).

**Fig. 1:**
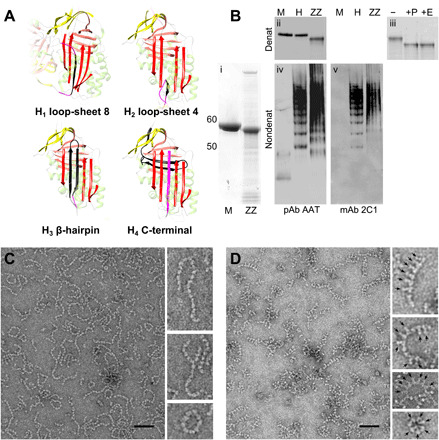
Characterization of α_1_-antitrypsin polymers present in patient explant liver tissue. (**A**) Different linkages hypothesized for the pathological polymer, H_1_ to H_4_, with the intermolecular interface proposed between one monomeric subunit and the next shown in black. (**B**) (i) Analysis of polymers isolated from intrahepatic inclusion bodies (denoted as ZZ) by 4-12 (w/v) acrylamide SDS-PAGE in comparison with the monomeric “wild-type” (M) variant purified from human plasma and visualized by Coomassie blue R stain. (ii, iv, and v) Western blots of ex vivo polymers (ZZ), polymers of the M variant induced by heating (H), and monomeric M variant (M) separated by denaturing SDS-PAGE (top) and nondenaturing native PAGE (bottom) and probed with a conformation-insensitive rabbit polyclonal antibody (pAb AAT, left) or a mouse monoclonal selective for polymeric α_1_-antitrypsin (mAb 2C1, right). No monomer is visible by native PAGE in the heat or ZZ preparation. (iii) Sensitivity of ex vivo Z α_1_-antitrypsin to PNGase F (+P) or EndoH (+E), the latter preferentially cleaving high-mannose glycans. (**C**) Representative micrograph of polymers isolated from ex vivo liver tissue, visualized by 2% (w/v) uranyl acetate negative stain using a Tecnai 120-keV transmission electron microscope at a magnification of ×92,000. The image has been low-pass–filtered to 30 Å. Black scale bar, 50 nm. Details of some polymers are shown at the right. (**D**) Same material, labeled with the Fab fragment of the 4B12 monoclonal antibody (Fab_4B12_), and visualized under the same conditions. Scale bar, 50 nm. Details from micrographs are shown at the right; readily discernible Fab protrusions are highlighted by arrows.

The structure of the pathological polymers that accumulate in patients has not been demonstrated. The observation that α_1_-antitrypsin polymers show a similar degree of stabilization to the cleaved form (*3*) (fig. S1B, EI) and that peptide analogs of the inserted portion of the reactive center loop (RCL) could similarly stabilize the protein (*4*) and prevent polymerization (*1*, *3*) suggested that polymers were the product of an interaction between the RCL of one molecule and β sheet A of the next (*1*). This “loop-sheet” model (Fig. 1A, hypotheses H_1_ and H_2_) is consistent with nuclear magnetic resonance and H/D (hydrogen-deuterium) exchange data showing that polymerization proceeds via a compact, rather than an expanded, intermediate (*5*, *6*). The subsequently proposed “β-hairpin” hypothesis (Fig. 1A, H_3_) was based on the crystal structure of a self-terminating dimer of a homologous protein, generated artificially at low pH, and extrapolated to α_1_-antitrypsin using limited proteolysis and recombinant mutants with stabilizing disulfide bonds (*7*). The “C-terminal” model (Fig. 1A, hypothesis H_4_) posits that the C terminus fails to form properly in the donor molecule and is instead incorporated into an acceptor molecule, with latent-like self-insertion of the RCL providing the extreme stability found in polymers (*8*). This model is based on a crystal structure of a denaturant-induced circular trimer of recombinant disulfide-bonded α_1_-antitrypsin. The circular arrangement of subunits provides a rigid structure that is tractable for crystallography but reflects a minor component of the source sample that is not generally enriched in polymer preparations (*1*), although there is an absence of the latent conformation in humans that would be predicted to be a by-product of this mechanism (*9*).

The question remains unresolved as to which polymerization model, if any, describes a realistic organization of the pathological polymer. To address this issue, we have performed a structural characterization of polymers from explant liver tissue of individuals homozygous for the Z allele who had undergone orthotopic transplantation. This has allowed us to define structural limits on the pathological polymer and to critically evaluate the proposed models in this pathological context.

## RESULTS

### Extraction of α_1_-antitrypsin polymers from liver inclusions

Tissue samples were obtained from the explanted livers of individuals homozygous for the Z allele of α_1_-antitrypsin. After isolation of inclusion bodies, polymers released by sonication were found to contain a major component that resolved at ~50 kDa when dissociated and visualized by denaturing SDS–polyacrylamide gel electrophoresis (SDS-PAGE) (Fig. 1B, i). It was confirmed to be α_1_-antitrypsin by Western blot analysis (Fig. 1B, ii). The difference in migration with respect to monomeric material purified from human plasma (Fig. 1B, i and ii) was no longer observed following treatment with PNGase F or EndoH (Fig. 1B, iii). This is diagnostic for glycosylated material that has not undergone maturation in the trans-Golgi network and therefore has been retained by the cell. When visualized by nondenaturing PAGE, the protein migrated with a broad size profile with some discrete bands visible, it was reactive with the polymer-specific (*10*) monoclonal antibody mAb_2C1_, and it was free of detectable monomer (Fig. 1B, iv and v).

### Negative-stain electron microscopy of liver-derived polymers labeled with a monoclonal antibody fragment (Fab)

The liver-derived polymers were applied to carbon-coated copper grids and negatively stained with 2% (w/v) uranyl acetate; polymers could easily be distinguished in the resultant electron microscopy (EM) images by a “beads-on-a-string” appearance (*1*), with a curvature of the chain and an absence of branching (Fig. 1C). While some circular forms were present, in contrast to a small-angle x-ray scattering (SAXS) analysis of polymeric material produced in the cytoplasm of *Pichia pastoris* (*11*), most (~80%) were non–self-terminating with clearly separated termini.

Polymer subunits are ~50 kDa in size, their ellipsoidal shape has few distinct features that would aid orientation, and they are connected by linkages that appear flexible. These properties provide confounding factors to processing by single-particle analysis. To facilitate subsequent image processing, we doubled the effective size of the polymer subunits and introduced an orienting feature by labeling polymers with the antigen-binding fragment of the 4B12 monoclonal antibody (Fab_4B12_) (*12*). This antibody was selected as it recognizes all folded forms of α_1_-antitrypsin including the polymer, and the location of its epitope is well established (*12*–*14*).

Following the addition of Fab_4B12_ at a stoichiometric excess to the α_1_-antitrypsin subunits and removal of unbound material, the polymer sample was visualized using negative-stain EM (NS-EM) (Fig. 1D). Fab_4B12_-labeled polymer subunits demonstrated additional density visible as “tooth-like” protrusions (Fig. 1D, insets). On consecutive subunits, Fabs were, in general, present on the same side of the polymer chain, potentially indicating a preference of the angular relationship around the polymer axis. Conversely, opposing α_1_-antitrypsin–Fab_4B12_ orientations, which would report substantial orientational freedom around the intersubunit linkage, were observed only infrequently.

### Selection and classification of dimer particles

The heterogeneity and flexibility of ex vivo polymers make them unsuitable for crystallography. Modern protocols for single-particle reconstruction of three-dimensional (3D) objects using EM images enable us to explicitly address heterogeneity in samples, and we therefore sought to structurally characterize the pathological polymers using this technique. A NS-EM image dataset of Fab_4B12_-labeled polymers was compiled from 100 × 30-frame movies that had been collected using a DE-20 direct detector and a Tecnai 200-keV transmission electron microscope. Preliminary experiments indicated that polymer flexibility would represent a challenge for a single-particle reconstruction approach. Thus, a minimal segment required to investigate the linkage between monomers—a dimer of adjacent subunits—was chosen for the subsequent structural analysis.

The processing pathway for single-particle reconstruction is described in more detail in the Supplementary Materials and in fig. S2 and is summarized here. Initially, images of dimer particles were manually selected from regions of polymers that appeared by eye to be side views with relatively little curvature (fig. S2*b*) and divided into classes using the Class2D function of RELION (*15*). The class sums included dimers in which the subunits appeared as adjacent ellipses, and many subunits exhibited a protuberance with the characteristic narrow midriff present in Fab structures (fig. S2*d*). In some classes, these Fab_4B12_ subunits were poorly resolved, suggesting variability in rotation between adjacent subunits. Seven classes with well-defined Fab_4B12_ components were used as references for autopicking from the same set of micrographs; after removal of poorly defined components, this yielded ~100,000 230 × 230 Å particle images. This dataset, D_A,100K_, was found by 2D classification to be more diverse and less dominated by long-axis dimer views (fig. S2*f*). Later in the course of processing, a subset of 69,000 dimer images (D_B,69K_) was extracted from a 2D reclassification of the same dataset (fig. S2*k*).

### EM reconstructions reveal two intersubunit configurations

One class in particular showed two well-resolved Fab subunits (fig. S2*h*). To generate an initial model-agnostic reference map for 3D classification, we converted this 2D image to a 3D surface representation (fig. S2*h*, right) with the height (along *z* in both directions) at each *x*,*y* coordinate proportional to the grayscale value of the corresponding pixel in the image (fig. S2*h*, right). This map was used as a reference for 3D classification of the D_A,100K_ dataset (fig. S2*i*). In two of eight resulting maps, both α_1_-antitrypsin molecules exhibited Fab_4B12_ protrusions. The best-defined map was divided in half, and one subunit was used as a monomer input reference in a reclassification of D_A,100K_ (fig. S2*j*). Following several iterations of 3D classification, five of eight classes exhibited either one or two well-defined α_1_-antitrypsin–Fab_4B12_ subunits (fig. S2*n*). These maps were divided in half, and the monomer subunits were individually superimposed and averaged together, providing a consensus density for the α_1_-antitrypsin–Fab_4B12_ monomer subunit Mon_av_ (fig. S2*o*, left). Mon_av_ was used as the reference map in successive rounds of 3D classification. Eventually, two classes were identified that showed connected α_1_-antitrypsin molecules with clear Fab_4B12_ subunits, comprising 9200 and 6200 particle images, respectively (fig. S2, *p* and *q*).

**Fig. 2:**
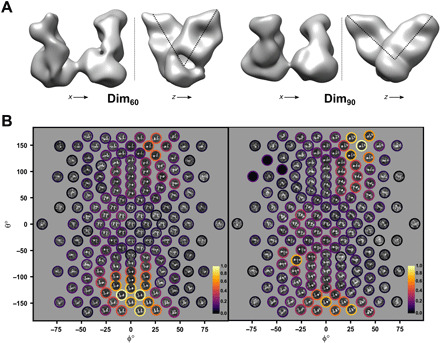
Reconstruction of monomer and dimer subunit density. (**A**) Orthogonal views of the reconstruction of Dim_60_ (left) and Dim_90_ (right) contoured at 3.9 × 10^5^ Å^3^. In this orientation, the connected α_1_-antitrypsin density is situated at the bottom, and the Fab domains are at the top. Calculated resolutions (using FSC = 0.33) are 19.1 and 24.8 Å, respectively (fig. S3). (**B**) Particle images, clustered by view and averaged, that are the basis for the reconstructions. The relative support for each cluster, calculated from the sum of the weights of the constituent images, is shown as circles colored according to a heatmap, highlighting the enrichment of views orthogonal to the dimer axis.

These 3D classes differed in the angles between the two α_1_-antitrypsin–Fab_4B12_ subunits—approximately 60° and 90°—and were accordingly termed Dim_60_ and Dim_90_ (Fig. 2A). Both showed clear Fab_4B12_ protuberances and connectivity between the volumes representing the α_1_-antitrypsin molecules. 3D refinement using “gold-standard” FSC (Fourier shell correlation) analysis provided estimated resolutions of 19.1 and 24.8 Å, respectively (at a FSC threshold of 0.33) (fig. S3). Other attempts to obtain dimer reconstructions using variations of the processing pathway described above also converged on these two forms and no others.

A summary of the constituent particle images, clustered by orientation relative to the 3D reconstructions, can be seen in Fig. 2B. In both cases, the assigned views show that the datasets contain a larger number of side-on views of the dimers, consistent with the observed alignment of most polymers in the micrographs.

### Heat-induced polymers

Polymers artificially induced at an elevated temperature have often been used to study the process of polymerization (*3*, *6*, *12*, *16*–*18*). It has been shown that this form shares a common epitope with ex vivo polymers in the vicinity of helices E and F (fig. S1A) (*14*); the epitope is not recognized when polymerization is artificially induced using a denaturant (*10*, *16*). The lack of discrimination between heat and liver polymers does not, however, demonstrate structural equivalence, and a means of direct comparison between the two has been lacking.

**Fig. 3:**
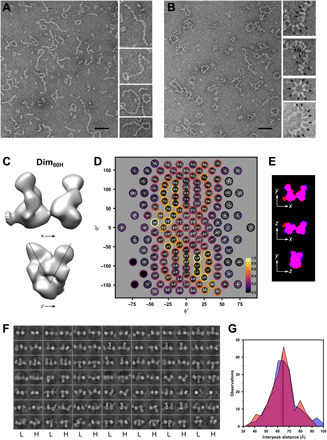
Characterization of heat-induced polymers. (**A**) Representative micrograph of polymers of M α_1_-antitrypsin induced at 55°C for 48 hours, visualized by 2% (w/v) uranyl acetate negative stain using the Tecnai 120-keV transmission electron microscope at a magnification of ×92,000. The image has been low-pass–filtered to 30 Å. Black scale bar, 50 nm. Details of selected polymers are shown at the right. (**B**) Heat-induced polymers labeled with Fab_4B12_ and visualized in the same manner. Details from micrographs are shown at the right; discernible Fab protrusions are highlighted by arrows. (**C**) Orthogonal views of the reconstruction of a Dim_60_-like structure, with a calculated resolution of 26.4 Å (FSC = 0.33) (fig. S3). (**D**) Particles upon which the reconstruction is based, clustered by imputed orientation and with the relative sum of their weights shown as a spectrum. (**E**) Orthogonal projections of the aligned and contoured Dim_60_ (blue) and Dim_60H_ (red) structures, with axes shown; overlapping regions appear as magenta. (**F**) 2D class sums from the liver and heat-induced polymer particle datasets arranged in pairs with columns denoted by L and H, respectively. For each liver polymer class, the most similar heat-induced polymer class by cross-correlation coefficient is shown; gray vertical lines through the images denote identified intensity peaks. (**G**) Distribution of the interpeak distances for the liver (blue) and heat (red) polymer distances. Dashed lines indicate the means of both sets of data.

Heat-induced polymers of the plasma-purified M variant were induced and purified, labeled with Fab_4B12_, and visualized by NS-EM using 2% (w/v) uranyl acetate stain. The resulting images showed the same flexible beads-on-a-string appearance (Fig. 3A), with a greater proportion exhibiting a circularized morphology. The Fab domains once again appeared as teeth-like protuberances with a general preferred orientation on the same side of the polymer axis in adjacent subunits with an occasional apparent ~90° to 180° inversion (Fig. 3B). A new dataset comprising 169 micrograph images was obtained, compiled from 30-frame movies collected using the DE-20 direct detector and the Tecnai 200-keV transmission electron microscope.

### Heat-induced polymers contain an enriched Dim_60_-type structure

We performed autopicking in RELION from the new micrographs using the same 2D references as with the ex vivo dataset (fig. S2*d*, right) because the heat-induced polymer subunits were of a similar size. Following rounds of 2D classification and cleaning of the image dataset, 25,000 dimer particles were extracted for further image analysis. In 3D classification, the monomeric subunit Mon_av_ (fig. S2*o*, left), obtained from the ex vivo dataset, was used as the reference; monomer rather than dimer was chosen to avoid introducing bias in the relative rotation and translation between subunits. At the final step of classification, a Dim_60_-type class was identified (Dim_60H_; Fig. 3C), comprising 6750 particles and with a nominal resolution of 26.4 Å (at FSC = 0.33; fig. S3). Clustering of particles by their orientation relative to the 3D volume again showed a preference for side views (Fig. 3D). Attempts at reclassification of the residual 18,000 particles failed to reveal further well-defined 3D classes.

### Comparison of liver-derived and heat-induced polymers

In a preliminary model-free analysis, the α_1_-antitrypsin–Fab_4B12_ dimer structure identified from the heat polymer data exhibited a somewhat different intersubunit distance and Fab_4B12_ orientation to that seen with the liver-derived dataset (Fig. 3, C and E): Translations and rotations of 64 Å/57° and 69 Å/65°, respectively, were required to superimpose a subunit volume onto the adjacent one. The correspondence more generally between the two datasets was therefore investigated. A comparison was made between all 2D classes obtained from the liver-derived polymer dataset against those calculated from the heat-induced polymer dataset by optimally aligning every possible pair and recording those with the highest correlation coefficient. Most pairs showed good visual correspondence (representative comparisons of class averages are shown in Fig. 3F). Positions of subunits were identified from peaks in the intensity profile of each image. The distribution of distances between these peaks in the aligned classes was almost identical, with a mean of 65 ± 12 and 64 ± 11 Å (±SD) for liver-derived and heat-induced polymer 2D classes, respectively (Fig. 3G). The putative distinction between the dimer volumes is therefore likely accommodated within the observed geometric relationships between subunits in both samples rather than supporting separate linkage mechanisms.

### An atomic model of the α_1_-antitrypsin–Fab_4B12_ subunit

**Fig. 4:**
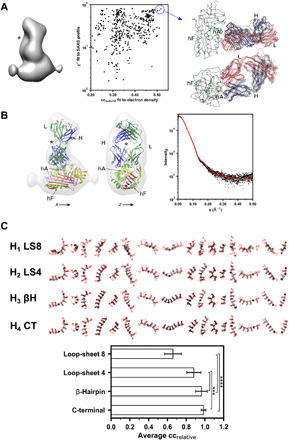
Atomic model of the α_1_-antitrypsin–Fab_4B12_ complex. (**A**) Left: Density for an α_1_-antitrypsin–Fab_4B12_ subunit calculated as the average of the Dim_60_ and Dim_90_ subunits, contoured at 1.9 × 10^5^ Å^3^ with a nominal resolution (at FSC = 0.33) of 15.2 Å (fig. S3). Middle: Result of modeling trials in which complexes between α_1_-antitrypsin and Fab_4B12_ molecules with random starting orientations were optimized with respect to the antibody epitope and the subunit density. The resulting structures were evaluated according to their correspondence with the experimental SAXS profile recorded for the complex. A cluster of structures maximizing both parameters are highlighted in red and circled. Right: Superposition on the α_1_-antitrypsin chain of these five structures showing a consistent relationship between the two components, with the heavy chain in blue and light chain in red. (**B**) Left: Final model of the subunit shown in the context of the experimental density, with the heavy chain in blue, the light chain in dark green, and α_1_-antitrypsin β sheets A, B, and C in red, pink, and yellow, respectively. The orientations are according to the axes shown in Fig. 3E. Right: Correspondence between the observed SAXS data (black) and the profile calculated from the coordinates of the final subunit model (red). (**C**) Top: Various polymer images extracted from NS-EM micrographs are shown in red, and 2D projections of polymer models that have been refined against these images are shown in black. Bottom: Mean relative correlations (±SD) between each model and the experimental density are shown. Values were calculated for each oligomer relative to the best score observed for that oligomer. Significance was determined by one-way analysis of variance (ANOVA) and Tukey’s multiple comparisons test (*n* = 18); ***P < 0.001 and ****P < 0.0001.

The 3D reconstructions of adjacent subunits reflect the asymmetric character of the Fab_4B12_-bound subunits and polarity of α_1_-antitrypsin within the polymer and embody shape, intersubunit distance, and rotational information. Accordingly, they could be used to challenge the different hypotheses regarding the structure of the pathological α_1_-antitrypsin polymer (Fig. 1A). As the foundation of this analysis, an atomic model of the Fab-antigen complex was required. Protein crystallization trials of Fab_4B12_ were successful and yielded a 1.9 Å structure, with the crystallographic parameters summarized in table S1. The asymmetric unit contained two molecules, one of which exhibited fully defined variable loop regions. Despite extensive efforts, it was not possible to obtain a crystal structure of the α_1_-antitrypsin–Fab_4B12_ complex; SAXS data were collected instead. The atomic model of the α_1_-antitrypsin–Fab_4B12_ subunit was then constructed using five sets of experimental data:

1) a consensus density map of the monomer generated by aligning and averaging the individual subunits of the Dim_60_ and Dim_90_ reconstructions from the liver polymer dataset (Mon_60,90_; shown in Fig. 4A, left);

2) the experimentally determined epitope of Fab_4B12_ (*13*, *14*) at α_1_-antitrypsin residues 32, 36, 43, 266, and 306 incorporated as a collection of distance constraints on the crystal structures of the individual components;

3) the Fab_4B12_ crystal structure;

4) the SAXS profile of the complex (Fig. 4B, right); and

5) the structure of cleaved α_1_-antitrypsin [Protein Data Bank (PDB): 1EZX (*19*)], as all extant polymer models propose a six-stranded β sheet A configuration (Fig. 1A).

Integration of these data during modeling was performed using PyRosetta (*20*). One thousand randomized starting orientations for α_1_-antitrypsin and Fab_4B12_ were subjected to rigid-body energy optimization with reference to these constraints and the Mon_60,90_ subunit map and scored according to both the cross-correlation coefficient (CCC) with the density and their correspondence with the SAXS profile (Fig. 4A, middle). Backbone and side-chain flexibility was conferred on regions of the Fab likely to contribute to the interface (heavy chain: 27 to 33, 51 to 57, 71 to 76, and 94 to 102; light chain: 27 to 32, 49 to 54, 66 to 70, and 91 to 94) and α_1_-antitrypsin side chains within the boundaries of the epitope.

The five models that maximized these metrics showed an unambiguous polarity (Fig. 4A, right). One model was selected that best represented this cluster by root mean square distance comparison with the others. This showed the heavy-light chain partition to be oriented off-center along helix A, with the variable-constant domain axis perpendicular to the long axis of the serpin [Fig. 4, A (right) and B (left)]. The cleft between the variable and constant domains of Fab_4B12_ aligned closely with a central “dimple” exhibited by the monomer density (denoted by an asterisk in the figure), and the complex corresponded well with the experimentally determined SAXS profile (Fig. 4B, right).

### Refinement of molecular models with respect to micrographs

**Fig. 5:**
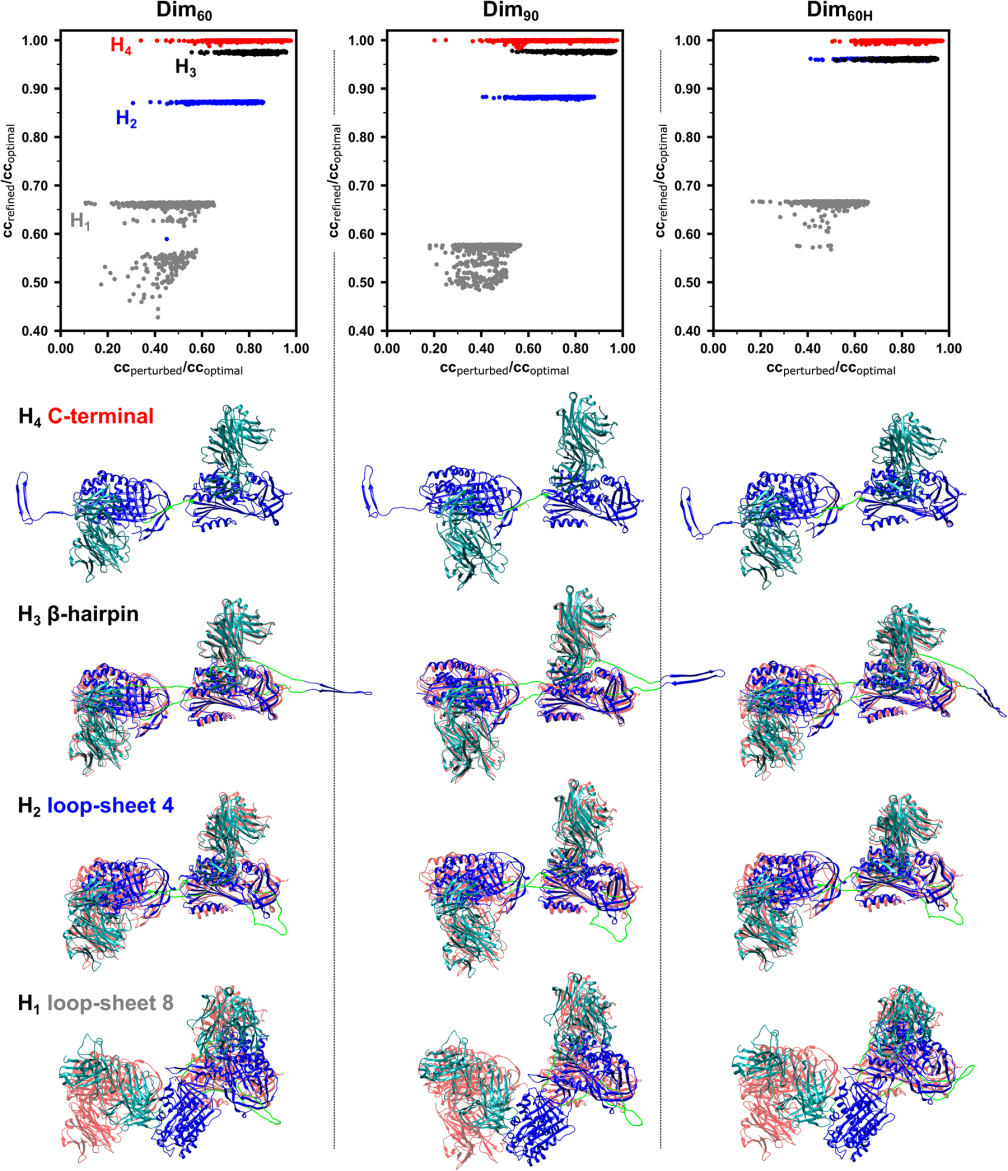
Optimization of the models of polymerization with respect to the experimental density. (**Top**) Different polymer configurations were randomly perturbed by rotation of the subunits with respect to one another and their conformations optimized against Dim_60_, Dim_90_, and Dim_60H_ reconstructions. The correlation coefficient after perturbation and before optimization is shown on the *x* axis, while that after optimization is shown on the *y* axis. Values are expressed relative to subunits optimized into the density without restriction by a connecting linker. Flexible regions encompassed residues 357 to 368 in all models as well as 340 to 349 (H_1_), 340 to 352 (H_2_), and 309 to 328 (H_3_). (**Bottom**) The best-fitting model for each polymer configuration and for each of the three dimer EM structures is shown (α_1_-antitrypsin in blue and Fab_4B12_ in dark green) with respect to the fit of unconstrained subunits (shown in pink). Regions treated as flexible linkers during the optimization are highlighted in light green. For all three reconstructions, the C-terminal model corresponds with the optimum arrangement of subunits.

Initial models of the C-terminal (*8*), loop-sheet (*1*), and β-hairpin (*7*) polymer configurations (Fig. 1A) were built using the α_1_-antitrypsin–Fab_4B12_ subunit structure (representations of these can be seen in the left column of Fig. 5), differing most substantially in the linker regions connecting adjacent subunits in the polymer chain (detailed in Materials and Methods).

From an examination of the representative micrographs shown in Fig. 1 (C and D), the intersubunit angular relationships along the polymer chains are not solely accounted for by the Dim_60_ and Dim_90_ configurations. Instead, these structures likely correspond to more highly populated species along a continuum of intermediate states. To investigate the compatibility of the loop-sheet, C-terminal, and β-hairpin linkages with the arrangement of polymers seen in the micrographs, we used a method that optimized the 3D models to maximize their correspondence with the 2D polymer images. Stretches of residues connecting the dimer subunits were treated as flexible (as specified in Materials and Methods), while the α_1_-antitrypsin–Fab_4B12_ cores behaved as rigid bodies. A selection of 20 oligomers was chosen with different degrees of curvature and subunit orientation (Fig. 4C). Despite a lack of information along the *z* axis, this approach was able to discriminate between the models on the basis of their ability to adopt the shapes seen in the 2D polymer images: The highly constrained loop-sheet eight-residue insertion model (H_1_) performed significantly worse than the others (*P* < 0.0001). The flexibility of the C-terminal domain swap (H_4_) provided a better fit than the loop-sheet four-residue insertion model (H_2_) (*P* < 0.001), and the β-hairpin (H_3_) and C-terminal models (H_4_) were not distinguishable by this analysis (Fig. 4D).

### Evaluation of models of intersubunit interactions with respect to the NS-EM structures

Next, the compatibility of loop-sheet, C-terminal, and β-hairpin configurations with the 3D Dim_60_, Dim_90_, and Dim_60H_ reconstructions was evaluated. Each model was repeatedly randomly perturbed by rotation around the dimer long axis (through the α_1_-antitrypsin subunits) and energy minimized with respect to the EM structures and default stereochemical restraints using PyRosetta (*20*). This process was undertaken 1000 times for each combination of model and map. As before, the α_1_-antitrypsin–Fab_4B12_ subunits were treated as rigid bodies connected by a flexible linker region. The correspondence between each model and the target map was assessed by the cross-correlation function. These CCC values were denoted as cc_perturbed_ and cc_refined_ for each perturbed model before and after energy minimization, respectively. Benchmark maximum CCC values were obtained by performing model-free alignments of α_1_-antitrypsin–Fab_4B12_ subunits into each map in the absence of a linker region and reported as cc_optimal_, denoted by red shaded models in the bottom panels of Fig. 5.

The result of this analysis is shown in Fig. 5 (top, color-coded by hypothesis). The random rotational perturbations applied to each model resulted in a spread of preminimization CCC values along the horizontal axis, and minimization of these models generally showed a convergence over a narrow range of CCC values on the vertical axis. The minimized structure giving the highest cc_refined_/cc_optimal_ score for each polymer configuration (in rows) with respect to each map (in columns) is shown in Fig. 5 (bottom). By this analysis, the best-scoring C-terminal polymers (H_4_) exhibited a value close to one, indicating that the linkage-restrained models were essentially indistinguishable from the unrestrained ones, and this was reflected by an almost direct superimposition of the model over the aligned linker-free subunits (top row). In contrast, the translational and rotational restrictions imposed by the linkers of the other models (H_1–3_) prevented them, to varying degrees, from adopting the preferred orientation inherent with respect to the data (bottom three rows).

All models entail a connection between β strand 4A of one α_1_-antitrypsin subunit and β strand 1C of the next. A distinguishing characteristic of hypotheses H_1–3_, with respect to the C-terminal model (H_4_), is that they involve a second unique intermolecular linkage. Having dual intermolecular constraints might be expected to reduce conformational flexibility, and this may contribute to their lesser compatibility with the density. To explore this, we performed a variation on the experiment in Fig. 5 in which the dual-linkage models were converted to single linkage by “breaking” the peptide bond between residues 358 and 359 of the β strand 4A-1C connection, leaving the unique second linker that each model embodies intact. During iterative rounds of optimization, displacement between residues adjacent to the site of cleavage confirmed that this modification allowed additional freedom of movement of the subunits. At the conclusion of the experiment, the scores obtained were very similar to those obtained with the intact models (fig. S4, top). We also performed the converse experiment, in which the β strand 4A-1C connection was kept intact, and the second unique linker of each model was broken (between residues 344 and 345 for H_1–2_ and 324 and 325 for H_3_). This provided comparable results to the single-linkage C-terminal model (H_4_) (fig. S4, middle).

These results demonstrate that the head-to-tail orientation of α_1_-antitrypsin subunits, with the base of β sheet A and the top of β sheet C in proximity to one another, is an intrinsic feature of the dimer density. Therefore, for the dual-linker models, it is not the reduced flexibility that distinguishes them but the inconsistency of their second linkage with this subunit orientation.

**Fig. 6:**
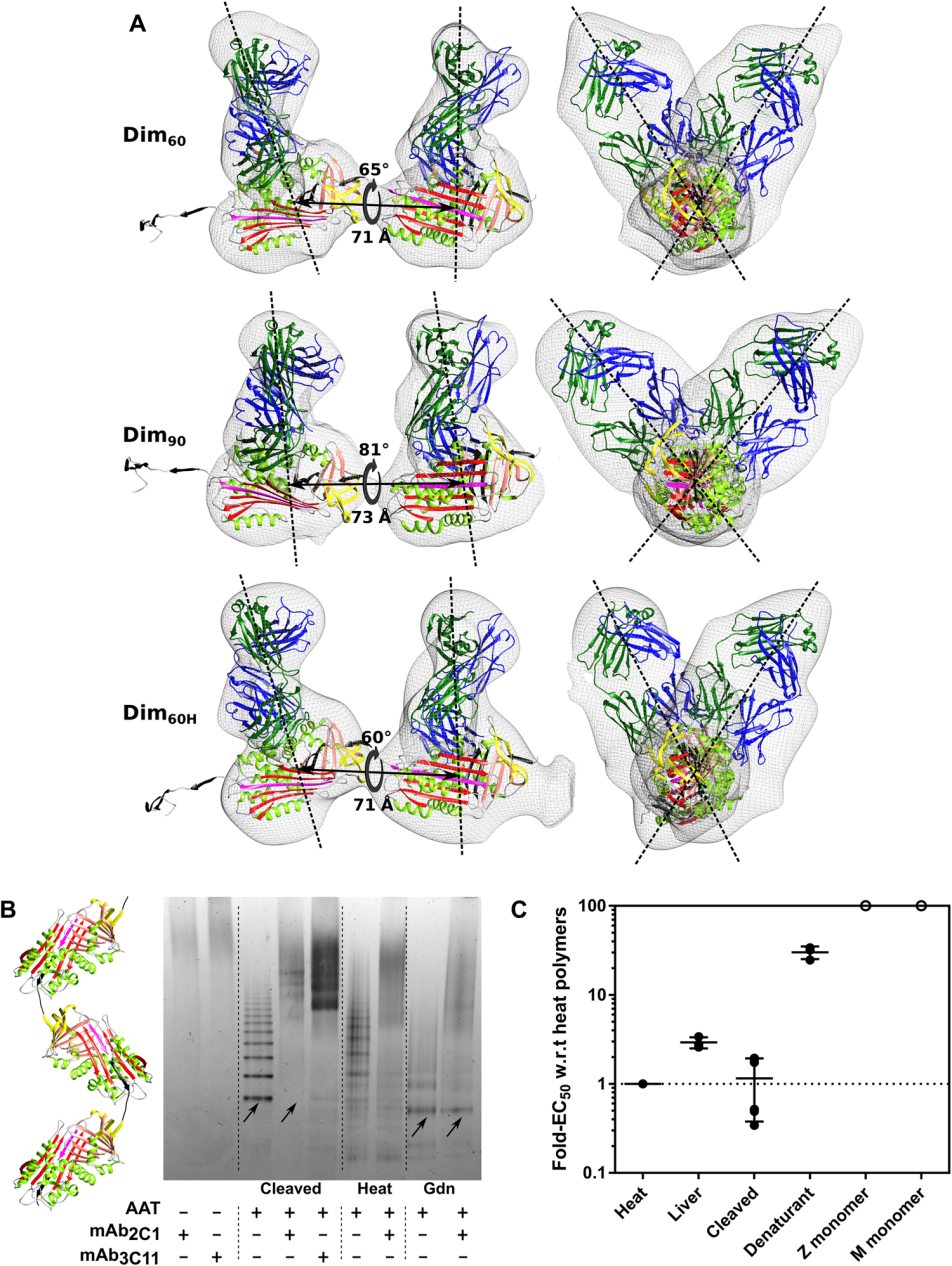
mAb_2C1_ recognizes structural analogs of C-terminal polymers. (**A**) Best-fitting C-terminal model (H_4_) displayed against the Dim_60_ (top), Dim_90_ (middle), and Dim_60H_ (bottom) density, annotated with intersubunit translations and rotations. Dashed lines represent vectors passing through the centers of mass of the α_1_-antitrypsin and Fab molecules. (**B**) Electrophoretic mobility shift assay comparing the affinity of the polymer-specific mAb_2C1_ for polymers of different origin. Binding of the antibody results in a cathodal shift of α_1_-antitrypsin polymers. Arrows highlight that cleavage-induced polymers, which are structurally analogous to C-terminal polymers, are readily recognized by the antibody with respect to denaturant-induced polymers. A schematic representation of P_9_-cleavage–induced polymers is shown at the left, with the domain-swapped peptide in black, based on PDB 1D5S (*21*). (**C**) Results of sandwich ELISA experiments showing the relative affinity of mAb_2C1_ for liver-derived, cleavage-induced, and denaturant-induced polymers, normalized to the half-maximal effective concentration (EC_50_) of the interaction with heat-induced polymers. The affinity of monomeric M and Z, denoted by open circles, was outside the maximum antigen concentration used in the experiment and, correspondingly, not less than two orders of magnitude worse than that of heat-induced polymers. Independent experiments are denoted by the markers, and the means ± SD are indicated by the bars (liver-derived and denaturant-induced, *n* = 3; cleaved, *n* = 6); heat-induced by definition is 1, represented by the dotted line; w.r.t, with respect to.

Thus, the orientation provided by the C-terminal model is most compatible with the Dim_60_ and Dim_90_ structures present in liver-derived polymers. In the final structure, there are translations of 71 and 73 Å between the centers of mass of the α_1_-antitrypsin molecules and a final calculated rotation around the dimer axis of 65° and 81°, respectively (Fig. 6A, top and middle). The same analysis, performed using the Dim_60H_ model derived from the heat-induced dataset, gave the same conclusion: The C-terminal model (H_4_) provided a fit consistent with the model-free aligned subunits (Fig. 6A, bottom). While there was a relative improvement in the fit of the loop-sheet 4 dimer, this model remained unable to adopt an optimal alignment to the experimental data (Fig. 5, right, and fig. S4, top right).

### The mAb_2C1_ antibody can recognize polymers in an open C-terminal configuration

A neoepitope is recognized by the mAb_2C1_ antibody that is present in liver-derived and heat-induced polymers but not in those induced in the presence of a denaturant. Thus, the latter conditions produce a polymer structure not representative of pathological material (*14*, *16*). Cleavage of the RCL of α_1_-antitrypsin in a noncognate position can also induce polymerization (*3*), and the atomic details of the resulting polymer linkage, defined by crystallography (*21*, *22*), show that it produces a molecule that mimics a noncircular form of the C-terminal trimer (*8*). To determine whether mAb_2C1_ recognizes the open C-terminal configuration identified from the EM analyses, polymers mimicking this structure were produced by limited proteolysis of a recombinant Ala350Arg α_1_-antitrypsin mutant by thrombin. This material was readily recognized by mAb_2C1_ as demonstrated in a mobility shift experiment (Fig. 6B). The relative affinity of mAb_2C1_ for the different forms was then determined by enzyme-linked immunosorbent assay (ELISA). These experiments exhibited comparable recognition of liver, heat-induced, and C-terminal–mimicking cleaved polymers by the antibody, with a markedly lower affinity for denaturant-induced polymers and monomer (Fig. 6C).

## DISCUSSION

α_1_-Antitrypsin deficiency is characterized by the accumulation of mutant protein as inclusions within hepatocytes. Extraction and disruption of these inclusions release chains of unbranched polymers, which, when isolated, exhibit pronounced flexibility and apparently lack higher-order interactions. Several models have been proposed for the molecular basis of the formation and properties of these polymers from in vitro experiments. On the basis of the observation that polymers are extremely stable and that artificially induced polymerization can be prevented by peptide mimics of the RCL, the first proposed loop-sheet molecular mechanism posited that the RCL of one molecule would incorporate into a β sheet of the adjacent molecule (H_1_ and H_2_ in Figs. 1A and 5) (*1*). Since that time, while biophysical studies have attempted to address the question of mechanism, the only crystal structures that have been obtained of α_1_-antitrypsin oligomers are of forms produced artificially from recombinant nonglycosylated material: a chain of molecules spontaneously assembled following fortuitous cleavage by a contaminating protease (*21*, *22*) and a circular trimer of a disulfide mutant produced by heating (H_4_) (*8*). Hence, there has been no direct evidence of the structure of the pathological polymers that deposit in the livers of patients with α_1_-antitrypsin deficiency.

The in vivo mechanism of α_1_-antitrypsin polymerization and accumulation in the liver has important consequences for the development of therapeutics that interfere with this process. The loop-sheet hypothesis (H_1_ and H_2_) involves relatively minor and reversible perturbations with respect to the native conformation to adopt a polymerization-prone state (*1*), the C-terminal model (H_4_) predicates a preceding substantial and irreversible conformational change (*8*), and the β-hairpin model (H_3_) lies somewhere between the two (*7*). This has implications for the nature of the site and mode of ligand binding capable of blocking polymerization and, indeed, for the question as to whether the process can be reversed at all.

Polymer material obtained from liver tissue is heterogeneous in size, glycosylated, and difficult to obtain in substantial quantity, making it unsuitable for crystallography. Without the requirement to form a crystal lattice, single-particle reconstruction using EM images represents an excellent option to obtain structural information. The negative-stain approach used here for the analysis of small protein complexes provided a strong contrast between protein and background and, in conjunction with decoration by Fab moieties, made angular information easier to retrieve, revealing the interactions between the components of the flexible polymer chains present in explant liver tissue.

Interrogation of the extant models of polymerization revealed that the loop-sheet dimer model (H_1_), despite its general compatibility with many biophysical observations, was unable to adopt the intersubunit translation or rotation observed in the 2D and 3D data (Figs. 4 and 5). A less stringent test of this model, a four-residue insertion loop-sheet configuration (H_2_) with an interchain interface analogous to one binding site of a tetrameric peptide blocker of polymerization (*23*), still provided an incomplete fit to the data. The β-hairpin domain swap model (H_3_), based on the structure of a self-terminating dimer of antithrombin, has been proposed to extend to α_1_-antitrypsin polymerization by limited proteolysis and the stability of a disulfide mutant against polymerization (*7*), a conclusion that has been questioned (*16*, *24*) and not supported by peptide fragment folding data (*25*). Owing to its longer predicted linking regions, the fit to the Dim_60_ and Dim_90_ data was better than that seen with the loop-sheet models (Fig. 5), but it required 20 residues to lose their native structure with respect to the antithrombin crystal structure from which this model is derived. While the crystal structure unequivocally demonstrates the ability of this form to adopt a 180° inversion orthogonal to the dimer axis, there was no evidence in the micrographs—either Fab-bound or unbound—of a chain inversion of this magnitude.

In contrast, the NS-EM data were best explained by the location, length, and flexibility of the C-terminal linkage (H_4_). The C-terminal mechanism involves displacement (or delayed formation) of the C-terminal 4-kDa fragment of α_1_-antitrypsin comprising β strands 1C, 4B, and 5B (fig. S1) and self-insertion of the RCL, which results in a monomeric latent-like intermediate conformation (*8*). The open, non–self-terminating arrangement of the subunits (Fig. 6A) contrasts with the observation that oligomeric components of recombinant material purified from *P. pastoris* were circular (*11*).

**Fig. 7:**
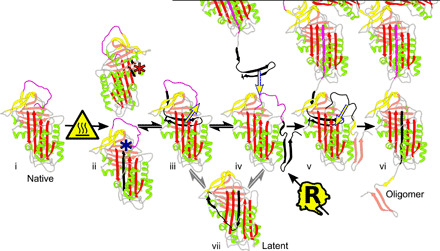
Putative model of the Z α_1_-antitrypsin polymerization pathway. From the native state (i), the evidence suggests that during heating, decreased affinity for the C terminus can be induced by destabilization of the adjacent breach region with increased solvation of the hydrophobic core (*26*, *27*), destabilization in the adjacent trihelix region (as in the S and I variants), and associated loss of strand 1C native interactions (ii and iii) (*6*, *24*, *32*). Upon dissociation of the C terminus, the molecule is equivalent to a final stage of folding of the nascent polypeptide chain (iv) (*25*). This (reversible) displacement is unable to immediately lead to self-insertion and generate the hyper-stable six-stranded β sheet A (*25*) despite delayed folding (*34*) (v), but such a change is able to proceed rapidly and irreversibly upon incorporation of the C terminus of another molecule (vi) (*25*, *33*). Under appropriate conditions, the latent conformation is generated as an off-pathway species (vii) that is expected to be inaccessible once full RCL insertion has taken place (v) (*17*, *36*). Asterisks denote Trp^194^ (blue) and Glu^264^/Arg^39^ (red), regions colored as black and yellow arrows highlight structural changes, and symbols indicate the application of heat (triangle) or a hypothesized point of convergence with the nascent chain folding pathway (“R”).

The data obtained, including the intersubunit orientation and distance (Figs. 3, F and G, 5, and 6A) and the presence of the mAb_2C1_ epitope (Fig. 6B), support a structural equivalence of heat-induced and liver-derived polymers. Hence, it follows that there will be components shared between their respective polymerization pathways; it should accordingly be possible to extend mechanistic observations made in vitro to the mechanisms that produce polymers in vivo, and here, we draw on observations made in the literature regarding the role of β strands 5A, 1C, 4B, and 5B and the breach region (Fig. 7). The ability to induce polymers from folded native α_1_-antitrypsin by displacement of the C-terminal region at modestly elevated temperatures in the Z variant implies that core packing interactions are readily destabilized when the molecule is in a five-stranded β sheet A configuration. In the native conformation (Fig. 7, i), the Z variant has been noted to increase the mobility of β strand 5A (*26*) and the solvation and rotational freedom (*27*) of the solvent-accessible (*28*) Trp^194^ residue that is situated in the breach region (Fig. 7, ii, bottom). The breach is bounded by a hydrophobic cluster of residues including some contributed by β strands 5A as well as C-terminal 4B and 5B, on which solvation (as reported by Trp^194^) would be expected to exert destabilizing effects. This is supported by sequential polypeptide folding experiments, suggesting that engagement of ~36 residues at the C terminus is predicated on a properly formed β strand 5A (*25*). A related process likely occurs on the opposing side of the molecule: Helices A, G, and H form a trihelix “clamp” over this region, and disruption of stabilizing interactions by the S (Glu264Val) and I (Arg39Cys) mutations (Fig. 7, ii, top) also leads to an increased tendency to polymerize upon the application of heat. Moreover, the fact that S, I, and Z are able to copolymerize (*29*, *30*) indicates that this occurs by a common mechanism and supports the mutual destabilization of the C-terminal region that is situated between them (Fig. 7, iii). This process is consistent with the site of polymerization-prone “latch” mutations clustered near the end of the polypeptide chain (*31*).

The early (*6*) and necessary (*24*, *32*) loss of native strand 1C contacts is consistent with the displacement of the C-terminal region (Fig. 7, iv). In this state, current evidence indicates that the molecule is equivalent to a final stage of the folding pathway (*25*). While the displaced C terminus (Fig. 7, iv) is relatively hydrophobic, in isolation, the equivalent “C36” peptide has been found to be soluble, albeit fibrillogenic over a period of hours, and readily incorporated into native α_1_-antitrypsin at room temperature, inducing an increase in thermal stability consistent with transition to a self-inserted form (*33*). This suggests that displacement of this region even at ambient temperature is possible. While, by analogy with release of the RCL by proteolytic cleavage (fig. S1), it might be expected that the release of the C terminus would immediately give rise to self-insertion of the untethered RCL as β strand 4A, there is evidence that the absence of an engaged C terminus will prevent this from occurring (*25*). This is congruent with the preferential folding of the protein to the kinetically stabilized five-stranded β sheet A conformation rather than the loop-inserted six-stranded thermodynamically favored state (*25*) despite the adoption of the hyperstable form upon administration of exogenous C-terminal peptide (*33*) and the fact that some material does fold correctly to the active form even with the delayed folding of the Z variant (*34*).

Upon incorporation of the C terminus of another molecule (Fig. 7, v), self-insertion of β strand 4A would be expected to follow (Fig. 7, vi) (*33*). The RCL of α_1_-antitrypsin is shorter than those of serpins known to undergo latency as a competing process to polymerization (*35*); once insertion has proceeded beyond a molecular “decision point” near the center of β sheet A (*17*, *36*), the molecule would no longer be able to (re-) incorporate its own C-terminal fragment (Fig. 7, vii), and it would effectively become irreversibly activated for oligomerization (Fig. 7, v). This mechanism is consistent with the suppression of polymerization in cells by a single-chain antibody fragment that alters the behavior of β sheet A in the vicinity of the helix F (*12*, *13*) and mutations that inhibit loop self-insertion (*17*).

Thus, of the proposed polymerization linkage models, our data most strongly support the C-terminal domain swap as the structural basis for pathological polymers of Z α_1_-antitrypsin. It remains to be determined how common or rare the exceptions are to this mechanism among other members of the serpin family. Serpins share a highly conserved core structure and exhibit common folding behaviors, and mutations that are associated with instability and deficiency tend to cluster within defined structural regions (*37*, *38*). These factors likely place constraints on the mechanism by which mutations can induce polymerization. It is difficult to overlook the central role of the C terminus in both latency and the C-terminal domain swap, with the former essentially a monomeric self-terminating form of the latter (Fig. 7, v to vii). While a shorter RCL likely renders these two states mutually exclusive in α_1_-antitrypsin, it has been suggested that the greater tendency of plasminogen activator inhibitor-1 (PAI-1) to adopt the latent conformation is due to a common origin in the polymerogenic intermediate (*35*). In support of this, PAI-1 and the neuroserpin L49P variant can form polymers from the latent state (*35*, *39*), a notable observation given the high stability of this conformation and inconsistent with the loop-sheet polymerization mechanism (which is predicated on a five-stranded native-like molecule) and the intermolecular β strand 5A/4A linkage of the β-hairpin model.

On the other hand, it has been shown that distinct alternative polymerization pathways are accessible in vitro depending on the nature of the destabilizing conditions used. The crystal structure of a β-hairpin–swapped self-terminating dimer of antithrombin (*7*) produced by incubation of this protein in vitro at low pH provides evidence of this. Similarly, induction of polymerization at acidic pH or with denaturants causes α_1_-antitrypsin to adopt a polymer form inconsistent with that seen upon heating or with pathological specimens from ZZ homozygotes (*16*). Biochemical evidence indicates that this may reflect the conformation of the rare α_1_-antitrypsin Trento variant (*14*).

From the data presented, here we expect the C-terminal domain swap to reflect the basis of pathological polymers in carriers of the Z α_1_-antitrypsin allele—and by extension, the S and I variants—and therefore account for more than 95% of cases of severe α_1_-antitrypsin deficiency. Because of its intimate association with the folding pathway and relationship with the latent structure more readily adopted by other serpins, it is probable that this form will be relevant to other serpin pathologies. Whether the same linkage underlies the shutter region mutants of α_1_-antitrypsin [such as Siiyama, Mmalton, and King’s (*2*, *10*)] that also cause polymer formation and severe plasma deficiency remains to be determined.

## MATERIALS AND METHODS

### Purified proteins

Human M and Z α_1_-antitrypsin were purified from donor plasma, and recombinant α_1_-antitrypsin was purified from *Escherichia coli* as previously described (*24*, *40*). Monoclonal antibodies were purified from hybridomas according to published methods (*12*) and stored in phosphate-buffered saline (PBS) with 0.02% (w/v) sodium azide. Fab fragments were generated by limited proteolysis using ficin or papain as appropriate with commercial kits according to the manufacturer’s instructions (Thermo Fisher Scientific) with the subsequent addition of 1 mM E-64 inhibitor.

### Isolation of ex vivo polymers

Explanted liver tissue (5 to 10 g) from individuals homozygous for the Z allele was homogenized and incubated at 37°C for 1 hour in 10 ml of Hank’s modified balanced salt solution with 5 mg of *Clostridium histolyticum* collagenase, and fibrous tissue was removed from the resultant suspension by filtration through BioPrepNylon synthetic cheesecloth with a 50-μm pore size (Biodesign). The filtrate was centrifuged at 3000*g* at 4°C for 15 min, the pellet was resuspended in 3 ml of 0.25 M sucrose in buffer E [5 mM EDTA, 50 mM NaCl, and 50 mM tris (pH 7.4)], and the sample was layered onto the top of two 14-ml centrifuge tubes (Beckman Coulter) containing a preformed 0.3 to 1.3 M sucrose gradient in buffer E and centrifuged at 25,000*g* for 2 hours at 4°C. The supernatant was discarded, and the pelleted inclusion bodies were washed with buffer E. Previous approaches to polymer extraction (*41*) have made use of detergents and denaturants, compounds that have been shown, under certain conditions, to induce conformational change in α_1_-antitrypsin (*3*), and therefore, we omitted their use. Soluble polymers were extracted by sonication on ice using a SoniPrep 150 with a nominal amplitude of 2.5 μm (giving a probe displacement of 17.5 μm) in bursts of 15 s and 15-s rest for a total of 6 min. The solution was repeatedly centrifuged for 5 min at 13,000*g* in a benchtop centrifuge to remove insoluble material. Purity of the soluble component was assessed by SDS- and nondenaturing PAGE.

### Preparation of artificially induced polymers

For heat-induced polymers, purified plasma M α_1_-antitrypsin was buffer-exchanged into PBS to 0.2 mg/ml and polymerization induced by heating at 55°C for 48 hours. Denaturant-induced polymers were formed by incubation at 0.4 mg/ml and 25°C for 48 hours in 3 M guanidine hydrochloride and 40 mM tris-HCl (pH 8) buffer. Following dialysis, anion exchange chromatography using a HiTrap Q Sepharose column with a 0 to 0.5 M NaCl gradient in 20 mM tris (pH 8.0) was used to remove residual monomer, as confirmed by native PAGE.

### Preparation of cleavage-induced polymers

An arginine residue was introduced at the P_9_ position (residue 350) of α_1_-antitrypsin in a pQE-30–based (Qiagen) expression system (*17*) using the QuikChange mutagenesis kit according to the manufacturer’s instructions (Agilent). Following purification from *E. coli*, the protein was subjected to limited proteolysis by a 50-fold substoichiometric concentration of bovine thrombin (Merck) at 37°C overnight and polymer isolated by anion exchange chromatography using a HiTrap Q Sepharose column with a 0 to 0.5 M NaCl gradient in 20 mM tris (pH 8.0).

### EM and image processing

Polymers were incubated with a threefold molar excess (with respect to subunit concentration) of Fab_4B12_ (*12*) for 2.5 hours at room temperature and repurified by anion exchange chromatography as described above or dialyzed overnight at 4°C into buffer E using a 300-kDa molecular weight cutoff membrane (Spectrum). Copper grids (300 mesh, Electron Microscopy Services) were covered with a continuous carbon film of thickness ~50 μm and glow discharged for 30 s. Three microliters of the prepared sample at ~0.05 to 0.1 mg/ml concentration was applied to the prepared grids for 1 min before blotting. Samples were negatively stained for 1 min using 5 μl of 2% (w/v) uranyl acetate and blotted, and the staining step was repeated. For single-frame high-contrast micrographs, grids were visualized using an FEI Tecnai T12 BioTWIN LaB6 microscope operating at 120 keV, and images were recorded on an FEI Eagle 4K × 4K charge-coupled device camera under low-dose conditions (∼25 electrons Å^−2^) at an effective magnification of ×91,463 (1.64 Å per pixel) and a defocus range of 0.8 to 3.5 μm. Micrographs for single-particle reconstruction were recorded as averages of 30-frame, 30-frames/s movies using a Tecnai F20 field emission gun transmission electron microscope at 200 keV with a Direct Electron DE-20 direct detector at a calibrated ×41,470 magnification (1.54 Å per pixel) under low-dose conditions (~1 electron Å^−2^ per frame). Frames were motion-corrected using MotionCorr (*42*). Resulting images were corrected for the effects of the contrast transfer function of the microscope using CTFFIND3 (*43*). Micrographs with greater than 5% astigmatism were discarded. Manual particle picking was undertaken using EMAN (*44*). General processing scripts in Python made use of the EMAN2 (*44*), NumPy, SciPy, OpenCV, and Matplotlib libraries.

### Single-particle reconstruction

RELION v2.1 and v3.0.6 (*15*) were used for single-particle reconstruction including automated particle picking, 2D and 3D classification, and 3D refinement, with the final processing path described in detail in Results and fig. S2. In general, classification in RELION used a regularization parameter *T* = 2 and 25 iterations or 50 iterations where convergence of statistics was not observed to have occurred. Image boxes were 230 × 230 Å in size; for 2D processing, a mask diameter of 180 Å was used, and alignment was performed using an initial 7.5° interval with an offset search range of five pixels; for 3D processing, the mask diameter was 195 Å with a sampling of 15° and eight pixels; and 3D refinement used 195 Å, 7.5°, and five pixels, respectively. Masks were generated for 3D dimer references by contouring at ~3.8 × 10^5^ Å^3^ (or at noise), for monomer references at ~1.9 × 10^5^ Å^3^, and both with the addition of a 7-voxel/7-voxel hard and soft edge. A 30-Å low-pass filter was applied to the resulting masked volumes before classification or refinement. After obtaining the Dim_60_ and Dim_90_ structures, the subsets of particle images on which they were based were subjected to a reference-free stochastic gradient-driven de novo reconstruction in RELION (sampling 15° and two-pixel increments; 50 initial, 200 in-between, and 50 final iterations from 40 Å down to 20 Å). An equivalent model was returned in each case. Similarly, combining the two particle sets together and performing a 3D reclassification using the monomeric Mon_av_ reference (fig. S2*o*, left) effectively returned the same two models.

### Sample characterization by electrophoresis

Proteins were resolved under denaturing conditions by NuPAGE 4 to 12% (w/v) acrylamide bis-tris SDS-PAGE gels and under nondenaturing conditions using NativePAGE 3 to 12% (w/v) acrylamide bis-tris gels (Thermo Fisher Scientific). For visualization by Coomassie dye, typical loading was 1 to 4 and 0.1 to 0.4 μg for Western blot. Western blot transfer to a polyvinylidene difluoride membrane was undertaken using the iBlot system (Thermo Fisher Scientific) or by wet transfer (Bio-Rad), followed by these steps: soaking in PBS for 10 min; blocking for 1 hour at room temperature with 5% (w/v) nonfat milk powder in PBS; incubation with primary antibody (rabbit polyclonal at 0.8 μg/ml or mouse monoclonal at 0.2 μg/ml) overnight at 4°C in PBS with 0.1% Tween (PBST), 5% (w/v) bovine serum albumin, and 0.1% sodium azide; washing with PBST; incubation with secondary antibodies at 1:5000 to 1:10,000 in PBST with 5% (w/v) bovine serum albumin and 0.1% sodium azide; and development by Pierce enhanced chemiluminesence (Thermo Fisher Scientific) or fluorescence (LiCor).

### Sandwich ELISA

High-binding enzyme immunoassay microplates (Sigma-Aldrich) were coated with 50 μl per well of anti-polymer mAb_2C1_ (2 μg/ml) in PBS with incubation overnight at room temperature, washed once with distilled water and twice with wash buffer [0.9% (w/v) sodium chloride and 0.025% (v/v) Tween 20], and blocked for 1 hour with 300 μl per well of PBST buffer [PBS, 0.025% (v/v) Tween 20, and 0.1% (w/v) sodium azide] supplemented with 0.25% (w/v) bovine serum albumin at room temperature (PBSTB). After washing the plates, antigens in PBSTB were applied by 1:1 serial dilution at a final volume of 50 μl across the plate, incubated for 2 hours at room temperature, and washed. Fifty microliters of rabbit anti-human α_1_-antitrypsin polyclonal antibody (1 μg/ml) (DAKO) in PBSTB was added to each well, the plates were incubated for 2 hours at room temperature and washed, 50 μl of a 1:2000 dilution of goat anti-rabbit horseradish peroxidase antibody in PBSTB (without sodium azide) was added to each well, and the plates were incubated in the dark for 75 min at room temperature and then washed again. For detection, 3,3′,5,5′-tetramethylbenzidine substrate solution (Sigma-Aldrich) was added at 50 μl per well, the plates were incubated for ~7 min in the dark, the reaction stopped by adding 50 μl per well of 1 M H_2_SO_4_, and the absorbance was promptly measured at 450 nm in a SpectraMax M5 plate reader (Molecular Devices).

### Protein crystallography

For crystallization trials, protein was buffer-exchanged into buffer C [10 mM tris (pH 7.4), 50 mM NaCl, and 0.02% (w/v) sodium azide] and concentrated to 10 mg/ml. Broad-screen sitting drop approaches against commercially available buffer formulations (Molecular Dimensions and Hampton Research) were performed with 100-nl protein:100-nl buffer drops dispensed using a Mosquito robot (TTP LabTech) and equilibrated against 75 μl of buffer at 16°C with automatic image acquisition by a CrystalMation system (Rigaku). Hanging-drop screens were performed at 20°C with 1 μl of protein:1 μl of buffer equilibrated against 250 μl of buffer. Crystals mounted on nylon loops were briefly soaked in the respective crystallization buffer supplemented by 10% (v/v) glycerol ethoxylate or 10% (v/v) ethylene glycol before plunge-freezing into liquid nitrogen. Data collection was undertaken at the European Synchrotron Radiation Facility (ESRF) ID30B beamline (with enabling work at the Diamond I03 beamline). Data reduction, integration, scaling, and merging were performed using autoPROC (*45*); the structures were solved by molecular replacement using Phaser (*46*); model refinement was undertaken with PHENIX (*47*); and model visualization and building were performed with Coot (*48*).

### Small-angle x-ray scattering

Recombinant α_1_-antitrypsin was incubated at a substoichiometric ratio to Fab_4B12_ for an hour at room temperature, and excess Fab was removed by anion exchange as described above. After concentration of the complex to 10 mg/ml, 50 μl was applied to a Superdex 200 Increase 5/150 column (GE Life Sciences) at a rate of 0.3 ml/min in 30 mM NaCl and 50 mM tris (pH 7.4) buffer at the P12 BioSAXS beamline, European Molecular Biology Laboratory (EMBL) Hamburg (*49*). The x-ray scatter (λ = 1.24 Å) was recorded on a Pilatus 6M detector at 1 frame/s. The buffer baseline-corrected scatter profile was produced by integration over time points corresponding with elution of the complex from the size exclusion column using the ATSAS software package (*50*).

### Initial model generation

For initial working subunit and dimer models, Coot (*48*) and PyMOL (Schrödinger Software) were used to position crystal structures of α_1_-antitrypsin [PDB: cleaved, 1EZX (*19*); cleaved polymer, 1D5S (*21*)] or mAb_4B12_ (PDB: 6QU9) and modify chain boundaries, repair gaps, and improve stereochemistry of intermolecular segments. The initial β-hairpin and loop-sheet models (Fig. 1A, H_1–3_) were further optimized in PyRosetta (Fig. 1A) (*20*). Superposition of the model of the α_1_-antitrypsin–Fab_4B12_ complex onto the dimer was undertaken using PyMOL. Modifications had to be made to each model to reconcile observations made here and in recent studies:

#### H_1_ and H_2_

Loop-sheet models have been represented with various degrees of insertion of the donor RCL into the site of β strand 4A in the acceptor molecule. To explore the compatibility of this parameter with the flexibility and periodicity of the polymers visualized here, two forms were generated, one with a substantial eight-residue insertion (loop-sheet 8, H_1_) and one with a marginal interaction at the base of β sheet A based on the observation that tetrameric peptides are able to block polymerization and induce stabilization of α_1_-antitrypsin (loop-sheet 4, H_2_) (*18*, *23*). The loop insertion site is permissive of noncognate peptide residues; however, such out-of-register insertion has not been observed crystallographically for intra- or interprotein loop insertion. For the arrangements used here, inserted residues were maintained in register at their cognate positions as observed for the structures of the cleaved protein, cleavage-induced polymer (*21*), and the self-terminating dimer (*7*) and trimer (*8*).

#### H_3_

The hypothesized unwinding of helix I in the β-hairpin polymer has been challenged (*16*) and is inconsistent with the role of this element in the 4B12 epitope (*13*). The ability of Fab_4B12_ to bind to the ex vivo polymers is unequivocal from the images recorded here; thus, if the pathological polymer is reflected by the β-hairpin model, then helix I must remain intact.

#### H_4_

Contrary to a proposal that circular polymers are the predominant species (*8*, *11*), most of those extracted from liver tissue were observed to be linear. Accordingly, the C-terminal dimer was arranged in an open configuration through redefinition of the chain boundaries in the crystal structure of a cleavage-generated polymer (*21*).

### Definition of flexible linker regions

During optimization of Fab-bound α_1_-antitrypsin dimer models, the constituent subunits were treated as rigid bodies connected by flexible linker regions. As much intersubunit linker flexibility was allowed as possible while maintaining the integrity of the core α_1_-antitrypsin fold, consistent with serpin monomer and oligomer crystal structures and with the high stability of the polymer. Divergence from the canonical structure was permitted where this accorded with the characteristics of the model being tested and other experimental data. Specifically:

1) Although crystal structures of cleaved antitrypsin polymers (*21*, *22*), an antithrombin dimer (*7*), and antitrypsin trimer (*8*) all have an intact strand 1C, it has been shown that during the process of (heat-induced) polymerization, this element is labile (*24*, *32*). Accordingly, we allowed the residues of this element (362 to 368) to move in all models.

2) All models of polymerization, either structurally defined or modeled, propose a connection between the C terminus of β strand 4A and the N terminus of β strand 1C (residues 357 to 362). The evidence is that this is a region that lacks secondary structure: In the cleaved form, it is not part of strand 4A or strand 1C; in the native structure, it does not form polar contacts with the body of the molecule; and it forms an extended chain in the latent conformation (*36*). Thus, this was treated as a flexible region.

3) The β-hairpin model (H_3_) involves a connection between helix I of the donor subunit and β strand 5A of the acceptor. Limited proteolysis data were interpreted to support the unraveling of helix I in this polymer linkage, yet this is not a feature observed in the crystal structure of the antithrombin dimer on which the model is based (*7*), and this conclusion has been disputed (*16*). If the β-hairpin model is indeed representative of the polymers considered in this study, then helix I should be intact as it is integral to the epitope of the non–conformation-selective Fab_4B12_ that decorates them (*13*). Hence, the region 309 to 328 between helix I and β strand 5A was provided with full flexibility, which maintains the integrity of elements seen in the original crystal structure but allows all other linker residues to move.

4) All crystal structures exhibit an intact β strand 5A, and while there is evidence of some lability of this structural element in the native conformation of a Z-like Glu342Ala mutant, this is not shared by the wild-type protein (*26*). For the loop-sheet models (H_1–2_) that propose connections between β strand 5A of the donor subunit and β strand 4A embedded in the acceptor, all connecting residues between residues 340 to 348 (H_1_) and 340 to 352 (H_2_) were provided full torsional freedom during refinement.

### Optimization of 3D models with respect to 2D oligomer density

The selection of polymers was performed manually by visual inspection of micrographs, followed by automatic thresholding and excision of regions of interest from the individual polymer images. Where a region of interest contained more than one chain, the image was postprocessed to remove density not related to the polymer of interest. Starting models of each polymer configuration at an appropriate length were generated by permutation of a “seed” dimer structure according to the number of subunits in an oligomer. The PyRosetta application programming interface (*20*) was then used, in which the α_1_-antitrypsin–Fab_4B12_ subunits were treated as rigid bodies connected by flexible linker regions; a full-backbone centroid model was used in which each side chain was represented by a single pseudoatom. Following an initial rigid-body step to approximately align the model with the image, loose positional constraints were applied to subunits according to the polymer path determined during the manual selections from the micrographs. Angular relationships with respect to the underlying substrate plane were inferred according to the extent of the orthogonal Fab protrusion observable, from 90° (evidence of increased density along the *z* axis only) to 0° (full-length protrusion in the *XY* plane). A necessary simplification, resulting in an implicit minimization of the magnitude of the angular displacement between subunits, was that these would tend to orient away from the underlying carbon substrate. Refinement of these models used an energy term that sought to increase the correlation between the experimental reference image and a 2D projection of the target 3D molecule. Standard stereochemical, repulsive, and attractive terms, and loose positional restraints, were maintained throughout. Iterative refinement proceeded for a minimum of 10 steps of 25 iterations, following which convergence was deemed to have occurred when the root mean square deviation between prerefined and postrefined model was less than 0.05 Å. The score for a given model-oligomer pair was calculated as the ratio of the best correlation coefficient observed during the optimization of the model against the oligomer relative to the best score observed for any model against that oligomer image.

### Iterative optimization of subunit interactions against 3D density

For each dimer configuration—loop-sheet 8 (H_1_) or 4 (H_2_), β-hairpin (H_3_), and C-terminal (H_4_)—repeated (1000) rounds of optimization were undertaken from a starting model randomly perturbed by rotation around the dimer axis. Full-atom models were represented as rigid subunits connected by flexible linkers. Optimization (using PyRosetta) involved an alternating sequence of whole-dimer rigid body shift and torsional optimization into the experimental density. The scoring scheme used to steer the process involved default internal stereochemical, attractive, and repulsive terms as well as the correlation of the atomic configuration with the EM density, with relative weighting of these terms progressively adjusted during the iterative procedure. To avoid any contribution of the linker regions to the scores obtained, only the rigid core subunits were used in the calculation of the correlation coefficient with respect to the electron density. The van der Waals scoring term was monitored to exclude models where unresolvable clashes occurred. Structures were visualized using Chimera (*51*) and PyMOL (Schrödinger Software).

### Statistical analysis

Statistical analyses were performed using Prism 6 software (GraphPad, La Jolla, CA, USA). The significance of the difference in correlation between the 2D projections of the different polymer models and the polymer images in Fig. 4 was determined by a one-way analysis of variance (ANOVA) and Tukey’s multiple comparisons test; ***P < 0.001 and ****P < 0.0001. Mean values are reported throughout the text with ±SD or ±SEM, as indicated.

### Human tissue samples

Tissue was used with the informed consent of donors and in accordance with local Institutional Review Boards.

## Supplementary Material

abc1370_SM.pdf

## SUPPLEMENTARY MATERIALS

Supplementary material for this article is available at http://advances.sciencemag.org/cgi/content/full/6/43/eabc1370/DC1

## Appendix

View/request a protocol for this paper from *Bio-protocol*.
