# Supplementary material for: The structural basis for Z α1-antitrypsin polymerization in the liver
Source: Sci Adv. 2020 Oct 21;6(43):eabc1370. doi: 10.1126/sciadv.abc1370 (PMC7577719; doi:10.1126/sciadv.abc1370)
Supplement: abc1370_SM.pdf [file abc1370_SM.pdf]

Science Advances
AAAS

advances.sciencemag.org/cgi/content/full/6/43/eabc1370/DC1

Supplementary Materials for

The structural basis for Z α_1-antitrypsin polymerization in the liver

Sarah V. Faull, Emma L. K. Elliston, Bibek Gooptu, Alistair M. Jagger, Ibrahim Aldobiyan, Adam Redzej, Magd Badaoui, Nina Heyer-Chauhan, S. Tamir Rashid, Gary M. Reynolds, David H. Adams, Elena Miranda, Elena V. Orlova, James A. Irving*, David A. Lomas*

*Corresponding author. Email: j.irving@ucl.ac.uk (J.A.I.); d.lomas@ucl.ac.uk (D.A.L.)

Published 21 October 2020, *Sci. Adv.* **6**, eabc1370 (2020)
DOI: 10.1126/sciadv.abc1370

The PDF file includes:

Methods – additional detail of single-particle reconstruction from EM micrographs
Table S1
Figs. S1 to S4

Methods – additional detail of single-particle reconstruction from EM micrographs

Single particle reconstruction from negative stain electron micrographs

Initially, 8000 images of dimer particles were manually selected from regions of polymers that appeared by eye to be side views with relatively little curvature (Fig. S2). These images were separated into 50 classes using the Class2D function of RELION (*15*). The class sums included dimers in which the subunits appeared as adjacent oblong densities, and many subunits exhibited a protuberance with the characteristic narrow midriff present in Fab structures (Fig. S2*d*). Some of the classes showed two poorly resolved Fab_{4B12} subunits, suggesting variability in rotation between adjacent subunits. Seven distinct classes with well-defined Fab_{4B12} components were selected and used as references for auto-picking; this yielded approximately 170,000 230×230 Å particle images. The following 2D classification of the selected images into 50 classes showed this dataset to be more diverse and less dominated by long-axis dimer views (Fig. S2*f*). Removal of obvious 'junk' particle images and side-views with significant curvature or with only one subunit resulted in a dataset of 100,000 images of dimers ($D_{A,100K}$). One class in particular showed two well-resolved Fab subunits (Fig. S2*h*). Later in the course of processing, a subset of 69,000 dimer images ($D_{B,69K}$) was extracted from a 2D reclassification of the same dataset into 200 classes (Fig. S2*k*).

An initial 3D model for 3D classification

To generate an initial model-agnostic reference map for 3D classification, the 2D class with both well-resolved Fab_{4B12} subunits was converted to a 3D surface mirrored along the Z axis (Fig. S2*h right*): in this representation, the elevation of the surface (in both positive and negative directions along Z) was proportional to the greyscale value of each pixel in the image and the resultant object was provided with a constant internal density. Visual comparison with the atomic structures of a representative Fab fragment (PDB: 1FD9) and loop-inserted α_1-antitrypsin (PDB: 1EZX) positioned using Chimera (*51*) showed the shape of the density to be reasonable (Fig. S2*h right*) and a likely relative orientation of the subunits, for this class at least, in near-planar arrangement.

A preliminary consensus reconstruction of the α_1-antitrypsin-Fab_{4B12} subunit

This map (Fig. S2*h right*) was used as a 3D reference for classification of the $D_{A,100K}$ dataset into eight 3D classes (Fig. S2*i*). In two maps both α_1-antitrypsin molecules exhibited Fab_{4B12} protrusions. One class contained a better-defined single α_1-antitrypsin-Fab_{4B12} subunit; this subunit volume was isolated using Chimera (*51*) and used as a monomer input reference in a subsequent 3D reclassification of $D_{A,100K}$ (Fig. S2*j*). This in turn provided a better-resolved dimer of adjacent subunits, which was then used as a reference in the classification of a particle dataset, $D_{B,69K}$, that had been re-processed to remove further 'junk' particles (Fig. S2*m*).

In the subsequent round, five of eight classes now exhibited one or two well-defined α_1-antitrypsin-Fab_{4B12} subunits (Fig. S2*n*). These monomer subunits were individually extracted, superimposed and averaged together in Chimera, yielding a consensus density for the α_1-antitrypsin-Fab_{4B12} monomer subunit, denoted here as Mon_{av} (Fig. S2*o*, *left*).

EM reconstructions reveal two inter-subunit configurations.

After removal of 24,000 particle images constituting 3D classes lacking sufficient subunit definition, 45,000 remained. Mon_{av} was used as the reference map in three successive 3D classifications. Eventually two classes were identified that showed connected α_1-antitrypsin molecules with clear Fab_{4B12} subunits, comprising 9200 and 6200 particle images respectively (Fig. S2*p,q*). Reiterating this process with the remaining particles failed to yield any density of similar quality.

Table S1. Data collection and refinement statistics for Fab_{4B12}.

Wavelength	0.9763
Resolution range	63.29 - 1.9 (1.968 - 1.9)
Space group	P 21 21 21
Unit cell	79.3 105.1 105.1 90.0 90.0 90.0
Total reflections	438567 (44953)
Unique reflections	69787 (6898)
Multiplicity	6.3 (6.5)
Completeness (%)	99.9 (99.9)
Mean I/sigma(I)	9.75 (1.76)
Wilson B-factor	40.0
R-merge	0.0813 (0.7818)
R-meas	0.0890 (0.851)
R-pim	0.0356 (0.332)
CC1/2	1.00 (0.84)
CC*	1.00 (0.96)
Reflections used in refinement	69774 (6894)
Reflections used for R-free	3388 (326)
R-work	0.202 (0.292)
R-free	0.234 (0.325)
CC(work)	0.96 (0.85)
CC(free)	0.96 (0.73)
Number of non-hydrogen atoms	6892
macromolecules	6444
ligands	23
solvent	425
Protein residues	848
RMS(bonds)	0.003
RMS(angles)	0.66
Ramachandran favored (%)	97.61
Ramachandran allowed (%)	2.39
Ramachandran outliers (%)	0.00
Rotamer outliers (%)	1.54
Clashscore	3.99
Average B-factor	48.05
macromolecules	47.95
ligands	53.15
solvent	49.28

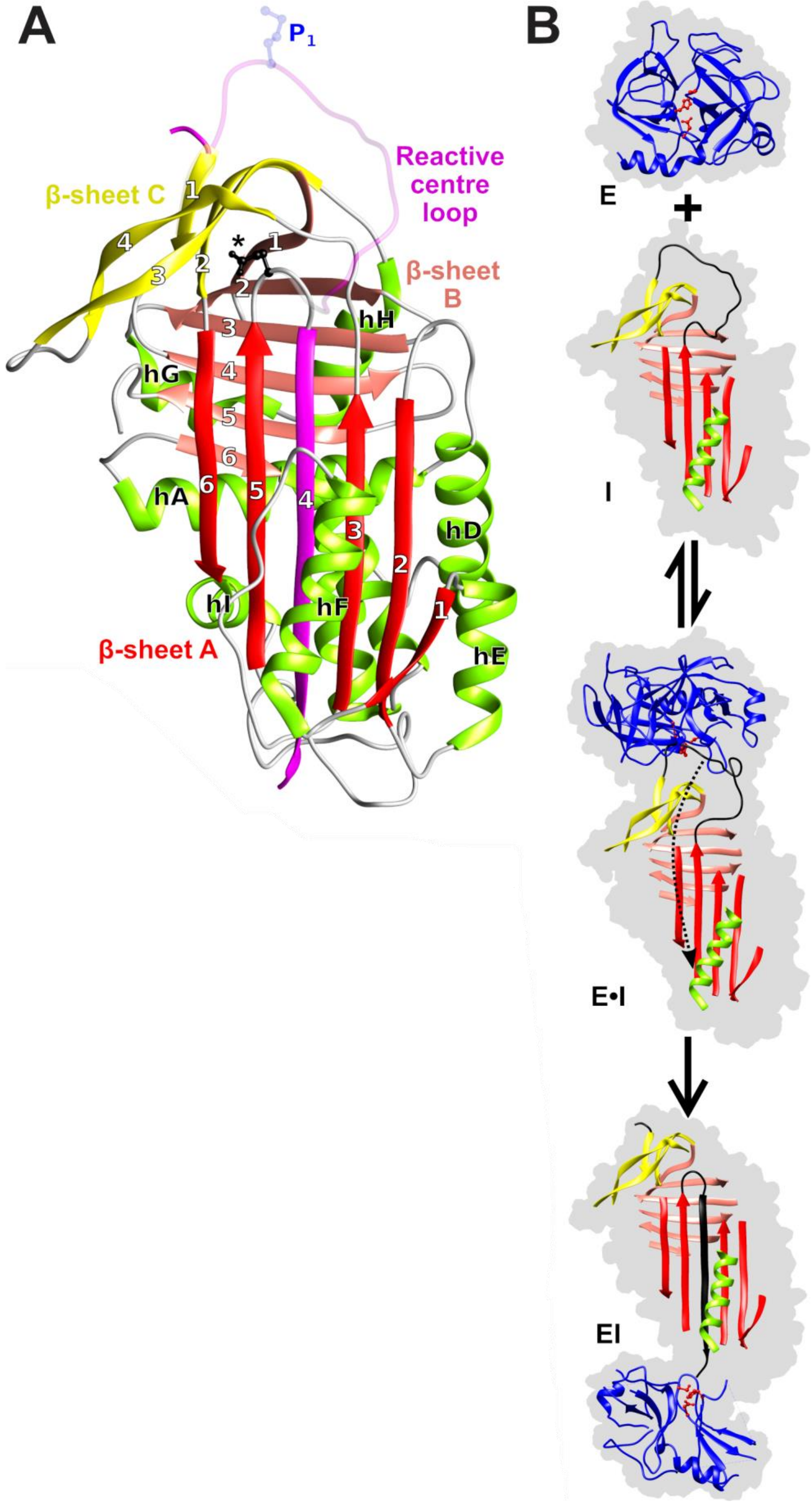

Fig. S1. Structure and conformational change in α_1-antitrypsin. (A) A schematic representation of α_1-antitrypsin (PDB: 1EZX) highlighting key structural elements. The protein is shown in the 'inserted' conformation; the exposed reactive centre loop (RCL; pale magenta) and the methionine residue at the position labelled P_1 in the native state are indicated by transparency for reference. These elements become incorporated into the central β-sheet A (red) as an extra strand (magenta) following proteolytic cleavage at P_1. Other elements indicated include β-sheet B in the core (salmon), β-sheet C (yellow) and helices in green. Numbers reflect β-strand designations. The site of the Glu342Lys (Z) mutation, in black ball-and-stick, is highlighted by an asterisk. **(B)** The mechanism of inhibition of α_1-antitrypsin. In its native state, a serpin has an exposed 'reactive centre loop' (RCL) and a 5-strand central β-sheet A, which represents a thermodynamically unstable conformation (I). The target protease, E, docks to a specific recognition sequence in the RCL to form a non-covalent 'Michaelis' complex, E•I. Cleavage of the RCL by the protease results in rapid insertion of the loop into β-sheet A, which translocates the protease by around 70 Å to the opposite pole of the protein. The result is a covalent complex, EI, in which the serpin is now in a thermodynamically hyperstable conformation and the protease catalytic machinery is maintained in a distorted, inactivated state (*19*). The montage was created using PDB structures 1QLP, 5MNP, 1OPH and 1EZX.

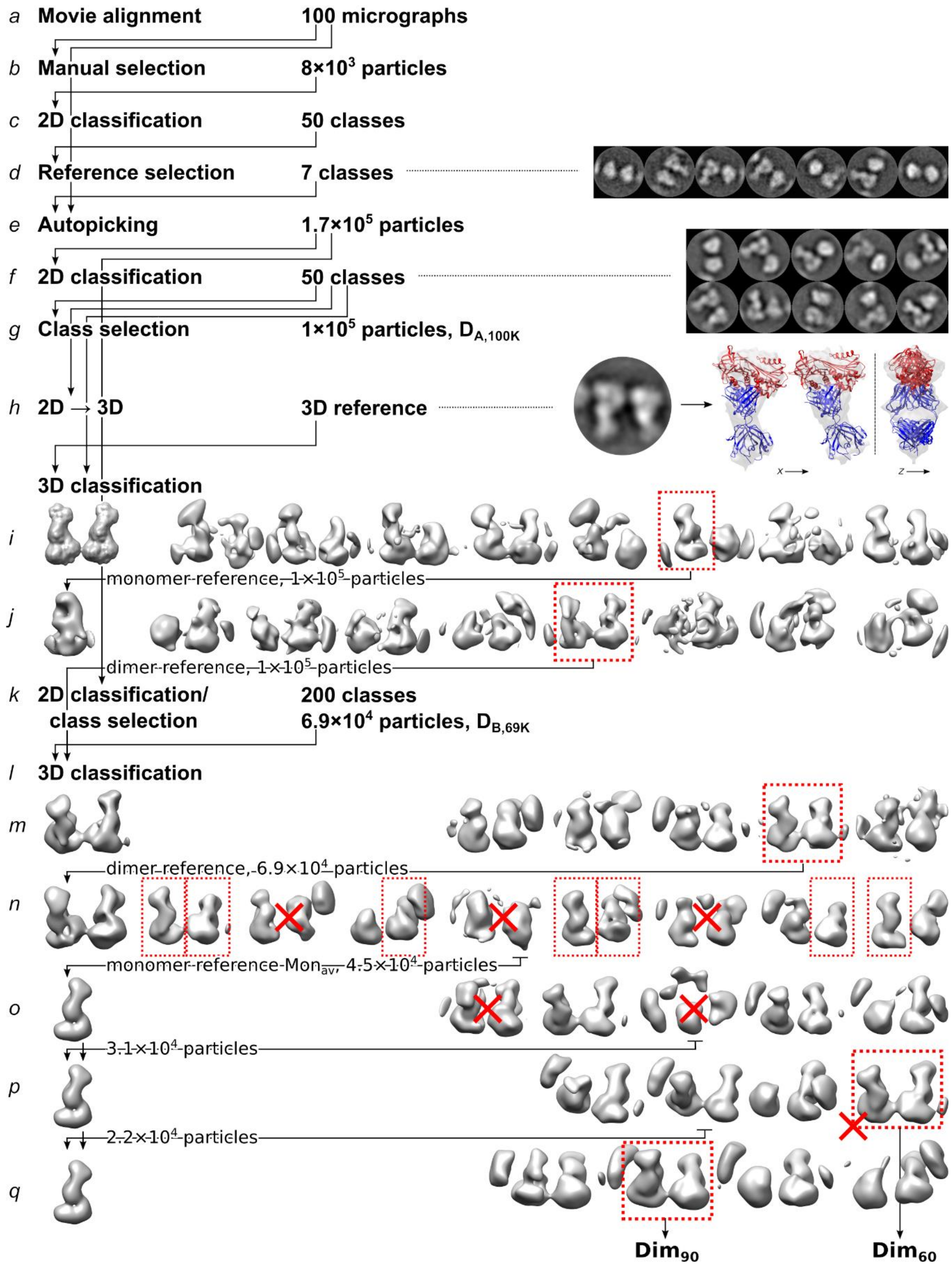

Fig. S2. The processing path resulting in the identification of two dimer structures. Single-particle reconstruction was largely undertaken in RELION (*15*), based on a dataset of 100 micrographs derived by alignment of 30-frame movies collected using a DE-20 direct detector on a Tecnai 200keV transmission electron microscope at 41,500× magnification. Pre-3D classification annotations denote processing (left), output (middle) and key detail (right). For 3D classification, the reference appears at the left and the output at the right. Classes selected for the construction of references in a subsequent iteration are denoted by red dashed boxes, and those whose constituent particles were removed from consideration are indicated by a '×'. Volumes are contoured at approximately 3.9×10^5 Å^3.

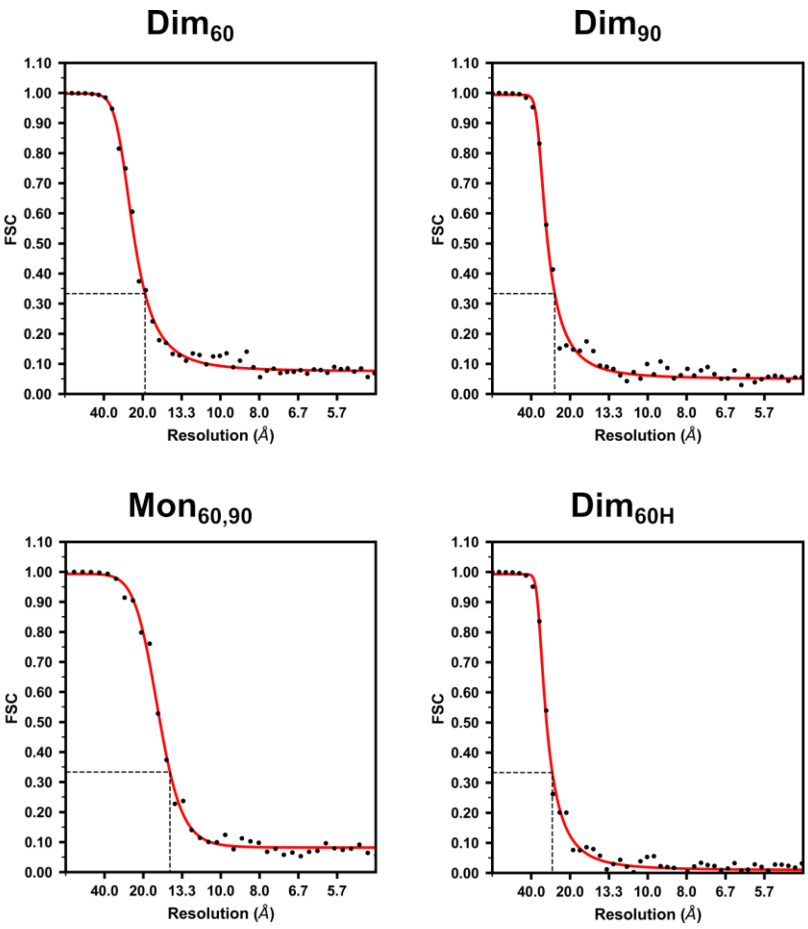

Fig. S3. Fourier shell correlation (FSC) analysis of NS-EM-derived structures. The particles used in each 3D reconstruction were randomly partitioned into two subsets and used in the semi-independent refinement of two 'half-maps' in RELION. Profiles were calculated from these half-maps using the PDBe Fourier shell correlation server at http://ebi.ac.uk. Dashed lines denote the resolution calculated at FSC=0.33.

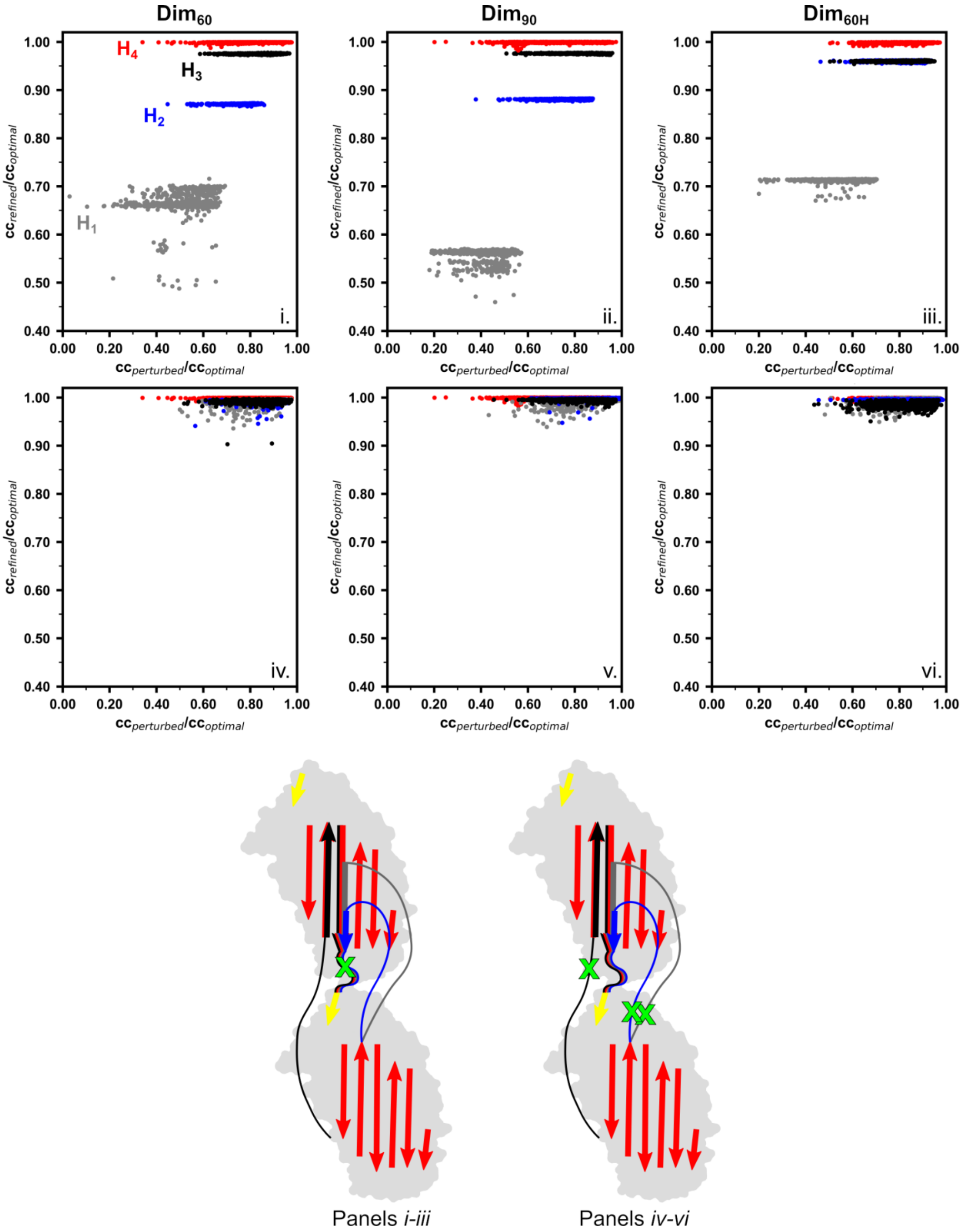

Fig. S4. Limits on potential mechanisms of polymerisation of α$_1$-antitrypsin. Dual-intermolecular linkage models of polymerisation (H$_{1\text{-}3}$) were converted to single-linkage forms and optimised with respect to the experimental density as performed for Fig. 5. In the *upper panels* (*i-iii*), the consensus linker between β-strand 4A of one subunit and β-strand 1C of the next was broken between residues 358-359. In the *middle panels* (*iv-vi*), the linker unique to each model was broken between residues 344-345 (H$_{1\text{-}2}$) and 324-325 (H$_3$). The *lower panels* summarise the experiments, with β-sheet A (red) and β-strand 1C (yellow) denoted by arrows, linkages coloured by model, and the green 'x' representing introduced chain breaks. The correlation coefficient after perturbation and before optimisation is shown on the X axis, while that after optimisation is shown on the Y axis. Values are expressed relative to subunits optimised into the density without restriction by a connecting linker. The values for single-linkage H$_4$ are identical to those in Fig. 5 and are shown for reference.
